# Adaptive evolution and reverse engineering to explore the low pH tolerance mechanisms of *Streptomyces albulus*

**DOI:** 10.1128/aem.00036-25

**Published:** 2025-03-31

**Authors:** Yuxi Liu, Tianyi Liu, Yulin Zhang, Liang Wang, Hongjian Zhang, Jianhua Zhang, Xusheng Chen

**Affiliations:** 1 Key Laboratory of Industrial Biotechnology, Ministry of Education, School of Biotechnology, Jiangnan University 546332, Wuxi, Jiangsu, China; Universita degli Studi di Napoli Federico II, Portici, Italy

**Keywords:** ε-poly-L-lysine, *Streptomyces albulus*, low pH tolerance, adaptive laboratory evolution, reverse engineering

## Abstract

*Streptomyces albulus* is well-known as a cell factory for producing ε-poly-L-lysine (ε-PL), but its ability to produce effectively requires an environment with a pH of about 4.0. Unfortunately, prolonged exposure to low pH environment compromises the cellular integrity of *S. albulus*, leading to a decrease in the efficiency of ε-PL biosynthesis. To enhance the low pH tolerance of *S. albulus* and investigate its low pH tolerance mechanisms, we employed adaptive laboratory evolution (ALE) technology to evolve the *S. albulus* GS114 strain by progressively lowering the environmental pH. This process ultimately yielded the mutant strain ALE3.6, which exhibited significantly improved low pH tolerance at pH 3.6 and achieved a 37.9% increase in ε-PL production compared to the parental GS114 strain under the optimal fermentation condition. The physiological evaluation of the mutant strain ALE3.6 indicated a pronounced enhancement in the integrity of its cell membrane and cell wall under low pH conditions. To identify the key genes involved in low pH tolerance, we employed whole-genome resequencing and quantitative real-time PCR, which pinpointed *desA*, *gatD*, and *mamU* as critical contributors. We further validated the roles of these genes through reverse engineering, which improved both low pH tolerance and ε-PL production efficiency. Finally, we elucidated the response mechanisms of the *S. albulus* cell membrane and cell wall under low pH stress. This study enhances the understanding of low pH tolerance in the *Streptomyces* species, particularly regarding the production of valuable biochemical products under challenging environmental conditions.

**IMPORTANCE:**

In this study, we improved the viability and ε-poly-L-lysine production efficiency of *Streptomyces albulus* at low pH by staged adaptive laboratory evolution while simplifying the previously studied fed-batch fermentation strategy. We identified key genes associated with the mutant strains’ cell membrane and cell wall phenotypes by utilizing whole-genome resequencing and reverse engineering. Subsequently, we validated the cell membrane and cell wall response mechanisms in *S. albulus* under low pH conditions.

## INTRODUCTION

*Streptomyces albulus* was first isolated from soil by Shima and Sakai in 1977 (1). Today, it is widely studied and utilized as an industrial strain for producing ε-poly-L-lysine (ε-PL) (2). ε-PL is a natural antimicrobial peptide composed of 25 to 35 L-lysine monomers, polymerized via an α-COOH and ε-NH_2_ condensation reaction, which is catalyzed by nonribosomal peptide synthetase (3). It exhibits potent inhibitory effects against a broad spectrum of microorganisms, including gram-positive and gram-negative bacteria, yeasts, molds, and viruses (4). This compound has diverse applications in the food industry, antimicrobial materials, pharmaceuticals, and animal feed (5). As a safe and effective natural preservative, ε-PL is approved for use in the food industry in regions such as Japan, the USA, and China (6). In the biomedical field, ε-PL has been incorporated into photopolymerized antimicrobial hydrogels, which serve as promising coatings for medical devices and implants due to their excellent biocompatibility and cell adhesion properties (7, 8). In animal husbandry, supplementation of ε-PL in animal feed can regulate the gut microbiota of Ningxiang pigs, enhancing the utilization of proteins, lipids, metabolic energy, and fiber (9). These diverse applications demonstrate the significant potential of ε-PL across various fields.

Previous studies have shown that *S. albulus* metabolizes inorganic nitrogen sources and secretes organic acids during ε-PL production (10). These activities cause the fermentation pH to gradually decrease from approximately 6.8 to around 3.0 during the natural fermentation process. The optimal growth pH for *S. albulus* is around 6.0, whereas the optimal pH for ε-PL production is approximately 4.0 (11). Prolonged exposure of *S. albulus* to low pH environments may compromise cellular integrity, ultimately leading to a decrease in the biosynthetic efficiency of ε-PL. To address this challenge, we have developed a dynamic pH-regulation strategy. This approach has successfully maintained the cell viability of *S. albulus* after acid pH shock while enhancing ε-PL production by 36.5% compared to fermentations that did not incorporate dynamic pH adjustments (12). However, we discovered that scaling up this process for industrial applications required cumbersome pH control, significantly increasing the difficulty of the fermentation process. Balancing process complexity with biosynthetic efficiency presents a substantial challenge, as it necessitates improving ε-PL synthesis efficiency per unit biomass under low pH conditions (13). Currently, research on the low pH tolerance mechanisms of *S. albulus* is relatively limited and lacks sufficient depth, which hinders the development of more efficient production strains. To address this challenge, it is urgent to develop low pH-tolerant strains and gain deeper insights into the low pH tolerance mechanisms.

In our previous study, we examined the response of *S. albulus* M-Z18 to low pH environments through transcriptomic analysis, providing a preliminary evaluation of its low pH tolerance system and the role of membrane fatty acid synthesis in low pH tolerance (13). Given that environmental acidification is inevitable during ε-PL biosynthesis in *S. albulus*, Ren et al. employed adaptive evolution in a medium with an initial pH of 4.0 to achieve acid tolerance through self-acidification (14). The resulting acid-tolerant mutants showed significant improvement in mitigating the effects of self-acidification on cell growth, ε-PL production, cellular respiration, and intracellular pH (pHi). Moreover, the upregulation of key genes confirmed the superior low pH tolerance of these mutants during self-acidification. Although the low pH tolerance of *S. albulus* has been improved through the process of self-acidification, there has been no significant improvement in ε-PL synthesis efficiency under low pH conditions. These improvements still have limitations in practical industrial applications. We speculate that previous adaptive evolution methods primarily focused on adapting cells to a pH of 4.0 (14), rather than progressively lowering the initial pH to achieve a higher degree of low pH tolerance. Evidence suggests that a gradual adaptation process can better acclimate microorganisms to new environments. For example, Zhang et al. developed mutant *Actinobacillus succinogenes* BC-4, which exhibits enhanced cell growth and improved succinic acid production under weakly acidic conditions through gradual adaptive evolution from pH 6.3 to pH 5.8 (15). Furthermore, although adaptive evolution has produced acid-tolerant strains, the underlying molecular mechanisms remain unclear. Therefore, genomic approaches should be employed to thoroughly investigate the low pH tolerance mechanisms of *S. albulus*. For example, Harden et al. conducted a genomic analysis of an *Escherichia coli* K-12 clone evolved under acidic conditions using resequencing technology. They identified acid adaptation mutations (*adiY* and *gcvP*), which may buffer pHi. These mutations significantly enhanced the clone’s adaptability at pH 4.6 (16).

In this study, we successfully obtained the mutant strain ALE3.6 by progressively lowering the initial pH of the growth medium, allowing *S. albulus* to adapt to increasingly low pH environments. The ALE3.6 strain exhibited rapid growth and efficient ε-PL synthesis under low pH conditions. Through whole-genome resequencing and quantitative real-time PCR (qRT-PCR) analysis, we identified several key genes associated with low pH tolerance, specifically *desA*, *gatD*, and *mamU*. Reverse engineering further elucidated the low pH resistance mechanisms of *S. albulus*, particularly in the cell membrane and cell wall, demonstrating that these genes enhance the strain’s pH resilience and ε-PL production efficiency. The study provides a novel foundation for improving the low pH tolerance of *Streptomyces* species for industrial applications and offers deeper insights into the underlying mechanisms of low pH resistance.

## RESULTS

### Adaptive laboratory evolution of *Streptomyces albulus*

**Fig 1:**
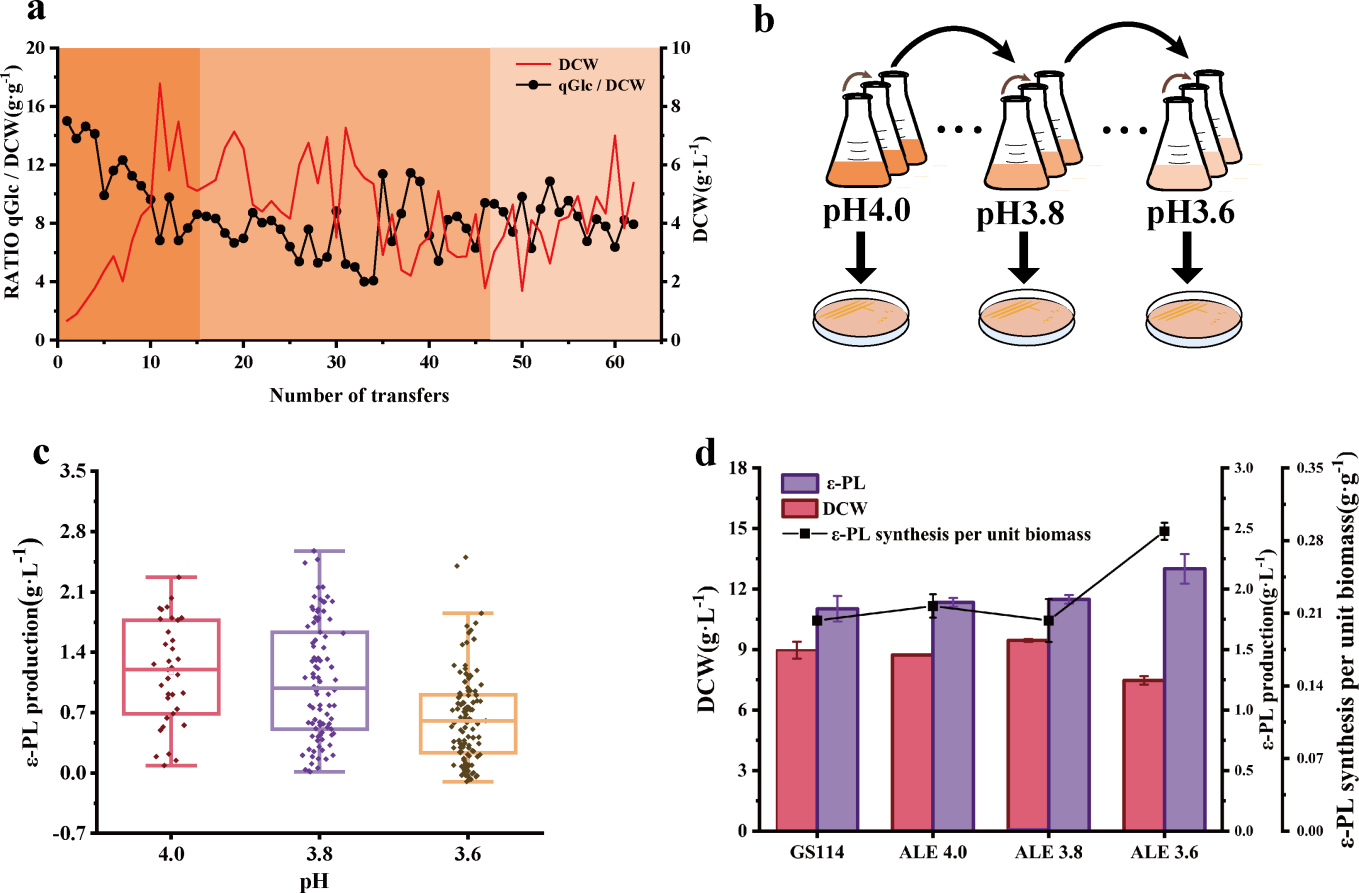
Adaptive evolution leading to the low pH-resistant mutant strain ALE3.6. (**a**) DCW and sugar consumption per unit cell during each of the 62 passages of adaptive evolution. (**b**) Schematic diagram of the gradual reduction of the initial pH during subculturing. (**c**) Screening and comparison of adaptive evolution isolates under different pH conditions based on ε-PL production. (**d**) Final DCW, final ε-PL production, and ε-PL synthesis per unit biomass of adaptively evolved strains ALE4.0, ALE3.8, and ALE3.6 compared with the parent strain GS114 after 96 h of shake-flask fermentation. Error bars indicate the standard deviations.

To enhance the low pH tolerance of *S. albulus*, low pH mutant strains were successfully isolated through stepwise adaptive laboratory evolution (ALE). During the ALE process, the strain underwent 62 continuous passages over 2,232 h (93 days), progressively adapting to a decrease in pH from 4.0 to 3.6 (Fig. 1a and b). However, when the adaptation pH was further lowered to 3.4, the lag phase was significantly extended, and growth ceased (data not shown). To ensure the improvement of low pH tolerance in *S. albulus* GS114, mutant strains were isolated at each pH stage and subjected to fermentation to compare their ε-PL synthesis capabilities. Subsequently, the three strains with the highest ε-PL production at each pH stage were selected and designated ALE4.0, ALE3.8, and ALE3.6 (Fig. 1c). Additionally, to further evaluate the low pH tolerance and production capacity of the mutant strains, a shake-flask fermentation was conducted. Compared to the parent strain GS114, the mutant strain ALE3.6 exhibited a 16.7% reduction in final dry cell weight (DCW). Despite this decrease in biomass, ALE3.6 achieved a 17.9% increase in ε-PL production and a significant 37.7% enhancement in ε-PL synthesis per unit biomass (Fig. 1d). Additionally, the final DCW of the mutant strain ALE3.8 was slightly higher than that of the parental strain GS114. However, statistical analysis showed no significant difference in the final DCW and ε-PL production between the mutant strains ALE4.0 and ALE3.8 compared to GS114 (Fig. 1d).

### ALE3.6 exhibited an enhanced ε-PL production capacity during fed-batch fermentation

**Fig 2:**
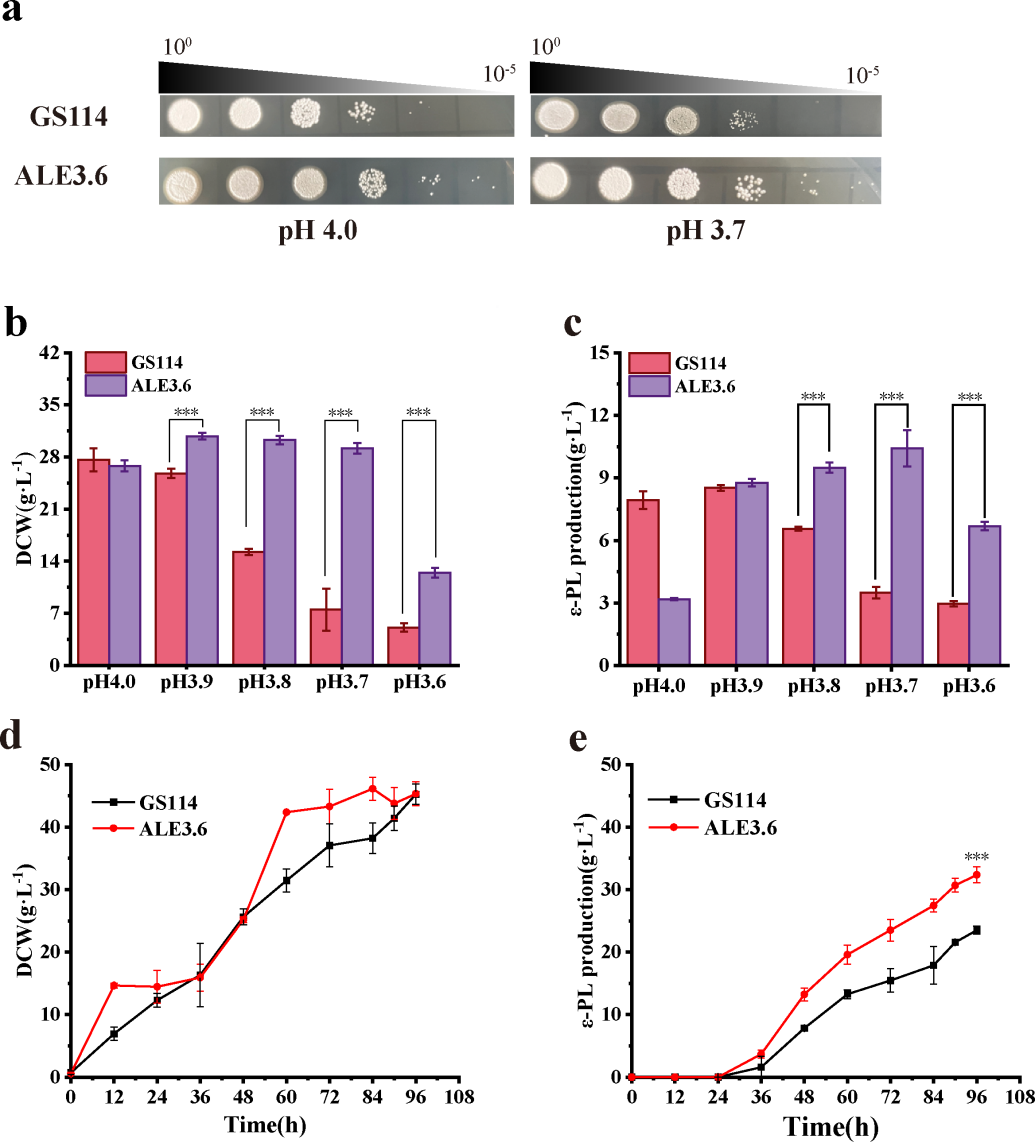
The mutant strain ALE3.6 showed an enhanced ability to synthesize ε-PL during fed-batch fermentation. (**a**) Spot assay of strains GS114 and ALE3.6 under pH 4.0 and 3.7 conditions. (**b**) Final DCW of strains GS114 and ALE3.6 after 60 h of fed-batch fermentation at constant pH 4.0, pH 3.9, pH 3.8, pH 3.7, and pH 3.6 conditions. (**c**) Final ε-PL production of strains GS114 and ALE3.6 after fermentation under the same pH conditions as in (**b**). (**d**) DCW of the parental strain GS114 and the mutant strain ALE3.6 under their respective optimal fermentation conditions. (**e**) ε-PL content of the parental strain GS114 and the mutant strain ALE3.6 under their respective optimal fermentation conditions. The experiments were performed in biological triplicate. Error bars indicate the standard deviations. ***, *P* ≤ 0.001.

To further confirm the low pH tolerance of the mutant strain ALE3.6, a spot assay showed that the spore growth of ALE3.6 was better than that of GS114 on a solid medium with a pH value of 4.0 (Fig. 2a). Unexpectedly, neither the parent strain GS114 nor the mutant strain ALE3.6 exhibited growth on a solid medium at pH 3.6. Consequently, we conducted a comparative analysis on solid medium at pH 3.7, where ALE3.6 showed significantly superior growth compared to the parent strain GS114 (Fig. 2a). Since the original fermentation condition (pH 4.0) used for adaptive evolution may not be optimal for ε-PL production by the mutant strain, we performed fed-batch fermentations for 60 h in parallel reactors under different constant pH conditions to compare the ε-PL production efficiency of GS114 and ALE3.6. The results indicated that, at pH 3.7, the final DCW of ALE3.6 reached 29.2 ± 0.7 g/L and the final ε-PL production reached 10.4 ± 0.9 g/L, which were significantly higher than those under other conditions (Fig. 2b and c). To evaluate the industrial application potential of ALE3.6, we conducted fed-batch fermentation for 96 h using the acid pH-shock fermentation strategy (17) (Fig. S2), adjusting the fermentation pH to 3.7 ± 0.1. Under the optimized pH condition, the ALE3.6 strain achieved the final DCW of 45.1 ± 1.6 g/L, the final ε-PL production of 32.4 ± 1.1 g/L, and ε-PL synthesis per unit biomass of 0.7 ± 0.01 g/g. In contrast, the parent strain GS114, which employed a dynamic pH-regulation strategy based on the acid pH-shock fermentation strategy under identical conditions, attained the final DCW of 45.3 ± 1.6 g/L, the final ε-PL production of 23.5 ± 0.7 g/L, and ε-PL synthesis per unit biomass of 0.6 ± 0.01 g/g. Therefore, ALE3.6 exhibited increases of 37.9% and 15.3% in the final ε-PL production and ε-PL synthesis per unit biomass, respectively, compared to GS114 under their respective optimal fermentation conditions (Fig. 2d and e).

### Adaptive evolution confers membrane stability to ALE3.6

**Fig 3:**
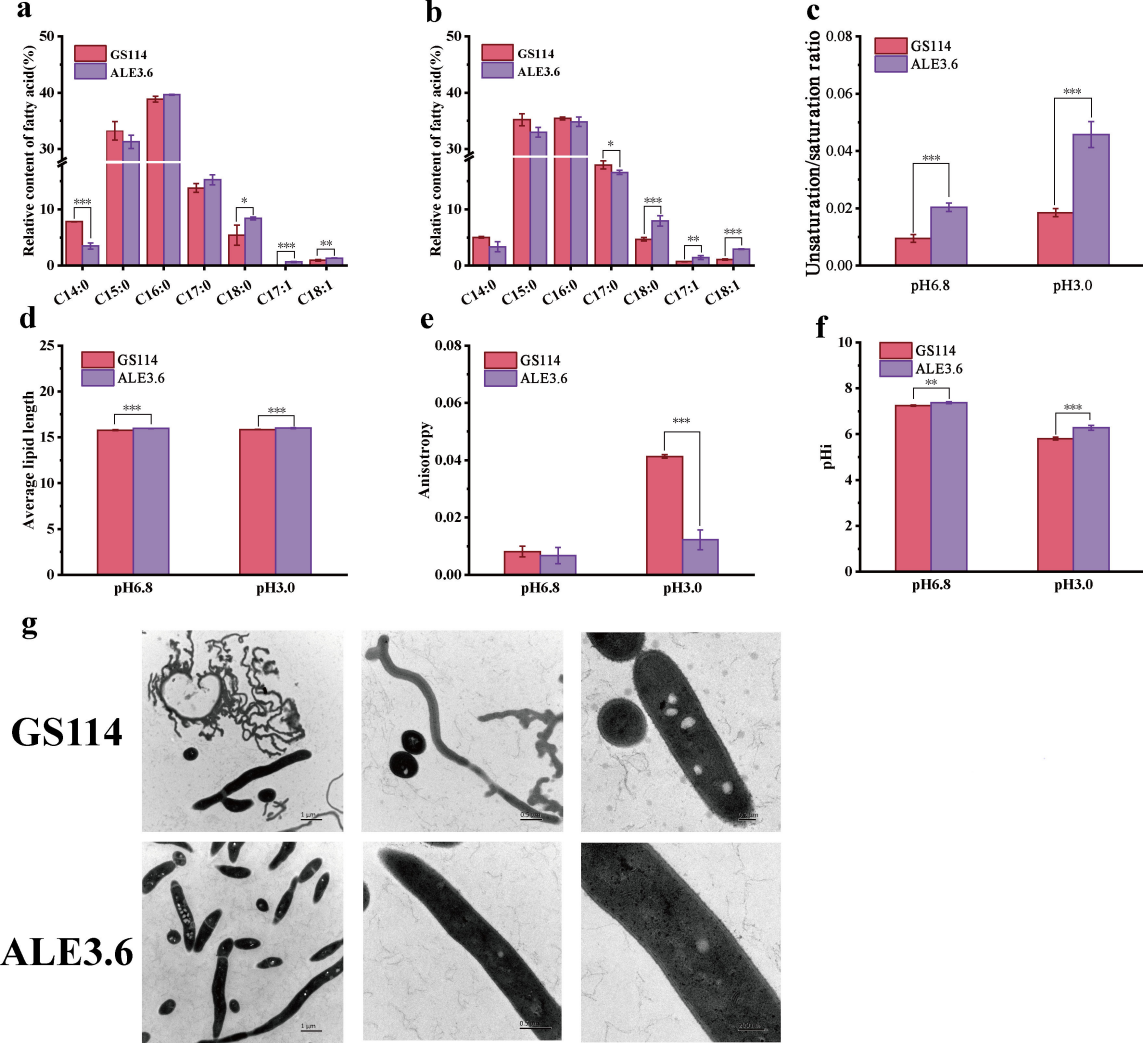
Adaptive evolution to low pH enhances tolerance of the *S. albulus* cell wall and membrane. (**a**) The fatty acid content in ALE3.6 and GS114 cell membranes after treatment at pH 6.8. (**b**) The fatty acid content in ALE3.6 and GS114 cell membranes after treatment at pH 3.0. (**c**) The unsaturation/saturation ratios in the cell membranes of ALE3.6 and GS114 after treatment under pH 6.8 and 3.0 conditions. (**d**) Average chain length of fatty acids in ALE3.6 and GS114 cell membranes after treatment at pH 6.8 and 3.0. (**e**) Effect of treatment under pH 6.8 and 3.0 conditions on membrane fluidity of strains ALE3.6 and GS114. (**f**) Intracellular pH of strains ALE3.6 and GS114 after treatment under pH 6.8 and 3.0 conditions. (**g**) Transmission electron microscopy images of strains ALE3.6 and GS114 after treatment under pH 3.0 condition. The experiments were performed in triplicate. Error bars indicate the standard deviations. *, *P* ≤ 0.05; **, *P* ≤ 0.01; ***, *P* ≤ 0.001.

To investigate the physiological changes of the mutant strain ALE3.6 under low pH conditions, we conducted a comparative physiological analysis between ALE3.6 and its parental strain GS114. Changes in the lipid content of bacterial cell membranes influence bacterial low pH tolerance (18). Therefore, we analyzed the fatty acid content in the cell membranes of ALE3.6 and GS114 using gas chromatography-mass spectrometry (GC-MS). At pH 6.8, the mutant strain ALE3.6 exhibited a significant 55.5% reduction in C_14:0_ fatty acid levels within its membrane compared to the parent strain GS114. In contrast, ALE3.6 showed substantial increases in C_18:0_ and C_18:1_ fatty acids by 54.7% and 42.6%, respectively. Additionally, a minor presence of C_17:1_ (0.66%) was detected in ALE3.6’s membrane, which was absent in GS114 (Fig. 3a). In order to simulate the lowest pH environment of natural acidification of *S. albulus*, we chose the initial pH of the culture medium to be 2.5, and the final pH after inoculation was about 3.0, so the subsequent low pH treatment conditions were all referred to as pH 3.0. At pH 3.0, the C_17:0_ fatty acid content in the membrane of mutant strain ALE3.6 decreased by 7.4% compared with strain GS114, while the C_18:0_, C_17:1_, and C_18:1_ contents significantly increased by 70.2%, 97.3%, and 168.8%, respectively (Fig. 3b). By analysis, the saturated:unsaturated ratio in the ALE3.6 membrane was significantly increased by 110.5% and 148.6% compared to GS114 at pH 6.8 and pH 3.0, respectively (Fig. 3c). Under these two conditions, the average chain length of cell membrane fatty acids in mutant strain ALE3.6 increased by 1.2% and 1.0%, respectively, compared with strain GS114 (Fig. 3d).

Since the content of unsaturated fatty acids in the cell membrane affects membrane fluidity (19), we used diphenylhexatriene (DPH) to measure the anisotropy of the cell membrane to detect the membrane fluidity of the strains. There is no significant difference between the membrane fluidity of ALE3.6 and GS114 under pH 6.8. After low pH treatment at pH 3.0 for 1.5 h, the membrane fluidity of the mutant strain ALE3.6 is surprisingly improved by 238.3% compared with GS114 under the same condition (Fig. 3e). Low pH-adapted bacteria maintain a higher pHi than the external environment, and membrane structural adaptations help to maintain better pH homeostasis (20). Therefore, we used the 2',7'-Bis-(2-carboxyethyl)-5(6)-carboxyfluorescein, acetoxymethyl (BCECF-AM) ester fluorescent probe to measure the pHi of the strain. After pH 3.0 treatment, the pHi of strain ALE3.6 is 6.28 ± 0.11, which is significantly different from the pHi of strain GS114 of 5.81 ± 0.06, with a *P*-value of less than 0.001 (Fig. 3f). To visually observe the changes in the mutant strain, we analyzed the cell morphology of ALE3.6 using transmission electron microscopy (TEM). After low pH treatment, the mutant strain ALE3.6 maintained its intact cell structure, while the cell wall of GS114 was relatively rough, and a large number of filamentous dead cells without intracellular structures were observed (Fig. 3g). These results indicate that the ALE3.6 strain exhibits more substantial membrane integrity and experiences less structural damage under low pH stress conditions.

### Resequencing analysis of ALE3.6

**Fig 4:**
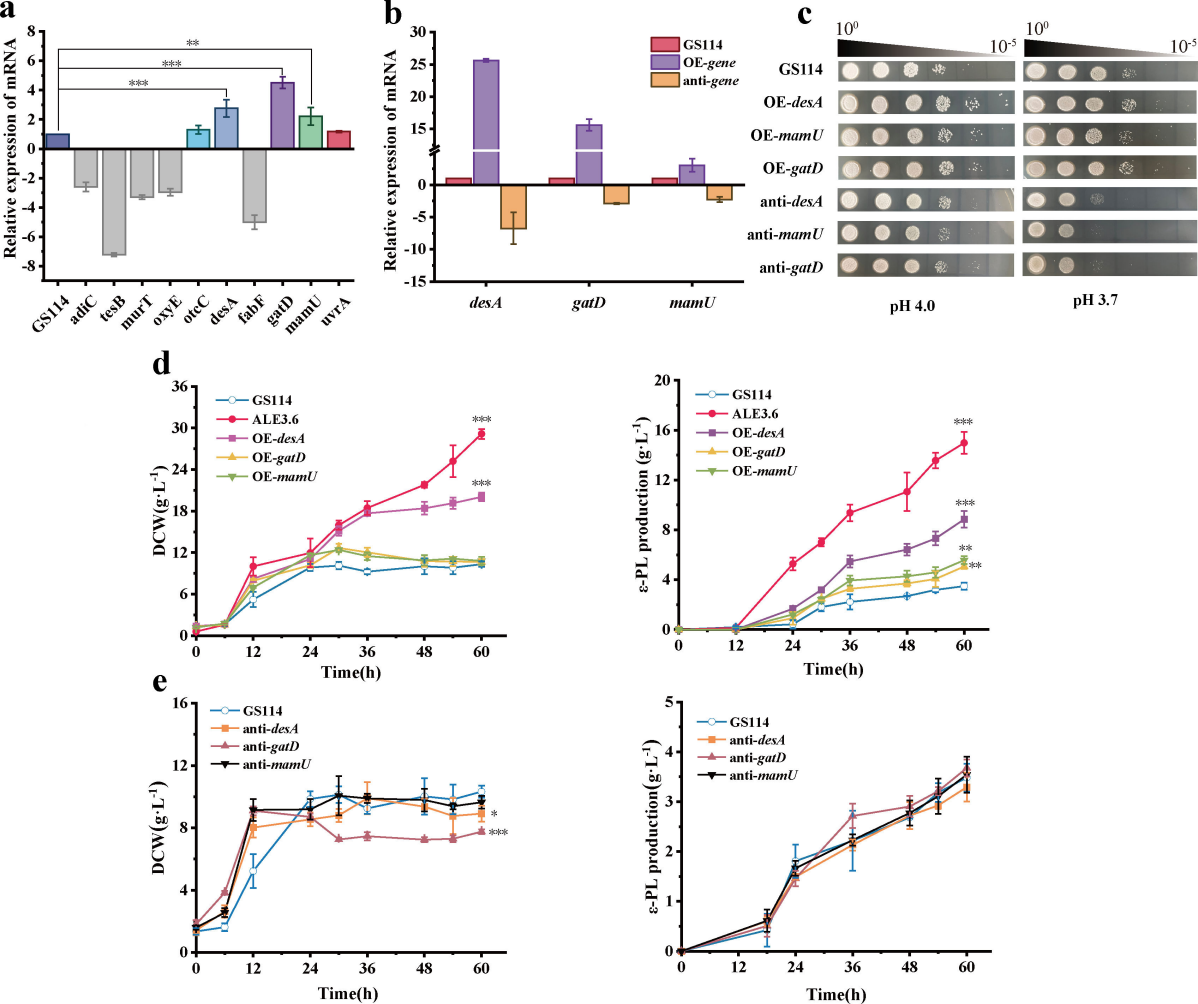
The key genes *desA*, *gatD*, and *mamU* enhance the low pH tolerance of *S. albulus* GS114. (**a**) Comparison of transcriptional levels of candidate genes in GS114 and ALE3.6 under low pH conditions. Error bars indicate the standard deviations. (**b**) mRNA expression levels of the corresponding genes in overexpressing and weakened strains relative to *S. albulus* GS114. (**c**) Growth performance of *S. albulus* strains with overexpression and weakening of the key genes (*desA*, *gatD*, and *mamU*) under low pH conditions at pH 4.0 and pH 3.7. (**d**) DCW and ε-PL production after 60 h of fed-batch fermentation at a constant pH of 3.7 for evolved strains and overexpressing strains (OE-*desA*, OE-*gatD*, and OE-*mamU*). (**e**) DCW and ε-PL production after 60 h of fed-batch fermentation at a constant pH of 3.7 for antisense RNA weakened strains (anti-*desA*, anti-*gatD*, and anti-*mamU*). All experiments were conducted in biological triplicate. Error bars indicate the standard deviations. *, *P* ≤ 0.05; **, *P* ≤ 0.01; ***, *P* ≤ 0.001.

To investigate the relationship between the genotype and phenotype of the mutant strain ALE3.6, we performed whole-genome resequencing of ALE3.6 and aligned the sequencing data to the reference genome of the parental strain GS114. We identified 22 single nucleotide polymorphisms (SNPs), 955 insertions and deletions (InDels), and 1,034 copy number variations (CNVs) (Fig. S1a). We found that strain ALE3.6 contained a large number of G and C base-related mutations in SNPs and InDels, but *S. albulus* already had a high G+C content; therefore, we currently consider these SNPs and InDels to be unrelated to the development of low pH resistance (Fig. S1b and c). However, our analysis has not found mutations associated with low pH tolerance in SNPs and InDels. So we focused on CNVs and found 10 mutations in the following genes that may be associated with low pH tolerance: amino acid permease AdiC (GS114GL005870), acyl-CoA thioesterase II TesB (GS114GL006362), small untranslated RNA OxyS (GS114GL006004), FAD-dependent oxidoreductase OxyE (GS114GL006647), FAD-dependent monooxygenase OtcC (GS114GL005997), putative acyl-[acyl-carrier-protein] desaturase DesA1 (GS114GL003573), beta-ketoacyl-[acyl-carrier-protein] synthase family protein FabF (GS114GL006580), MurT ligase domain-containing protein MurT (GS114GL006732), glutamine amidotransferase GatD (GS114GL006731), bifunctional phosphatase PAP2/diacylglycerol kinase family protein MamU (GS114GL005861), and excinuclease ABC subunit UvrA (GS114GL006279). To further link these CNV genes to the phenotype, we employed qRT-PCR to assess the expression levels of these genes in GS114 and ALE3.6 following low pH treatment. The results showed that under low pH conditions, the expression levels of *desA*, *gatD*, and *mamU* in ALE3.6 were significantly elevated compared to GS114 under the same condition (Fig. 4a).

### Key mutations enhance the adaptability of *S. albulus* to low pH fermentation

To assess whether *desA*, *gatD*, and *mamU* enhance low pH tolerance in *S. albulus*, we introduced the pIB139 plasmid into GS114 to enhance gene expression, generating strains OE-*desA*, OE-*gatD*, and OE-*mamU*. We also used recombinant plasmids carrying a 24 bp antisense fragment of the target gene to reduce its expression in GS114, creating strains anti-*desA*, anti-*gatD*, and anti-*mamU*. qRT-PCR was conducted to verify the expression levels of these genes under low pH conditions. The results confirmed that overexpressed strains achieved the expected increase in gene expression, while weakened strains attained the anticipated reduction (Fig. 4b). In the spot assay, the overexpressing mutant strains showed significantly better growth performance at pH 3.7 compared to GS114, while the antisense RNA strains displayed significantly reduced growth (Fig. 4c). Additionally, the final DCW of OE-*desA* was significantly increased by 104.0% compared to GS114 in a 60 h fed-batch fermentation at pH 3.7. However, OE-*gatD* and OE-*mamU* did not exhibit significant differences. The final ε-PL production of OE-*desA*, OE-*gatD*, and OE-*mamU* increased by 169.0%, 53.8%, and 69.3%, respectively, compared with GS114 (Fig. 4d). Under the same fermentation conditions, the final DCW of anti-*desA* and anti-*gatD* showed 13.8% and 25.0% reduction compared with GS114, while the DCW of anti-*mamU* did not change significantly. Unfortunately, anti-*desA*, anti-*gatD*, and anti-*mamU* did not significantly differ in final ε-PL production compared with GS114 (Fig. 4e). Although there was no significant difference in the final ε-PL production of the weakened strains, we can still preliminarily judge from other conclusions that *desA*, *gatD*, and *mamU* genes play a crucial role in conferring low pH stress resistance to *S. albulus* GS114.

### Key mutations maintain *S. albulus* stability

**Fig 5:**
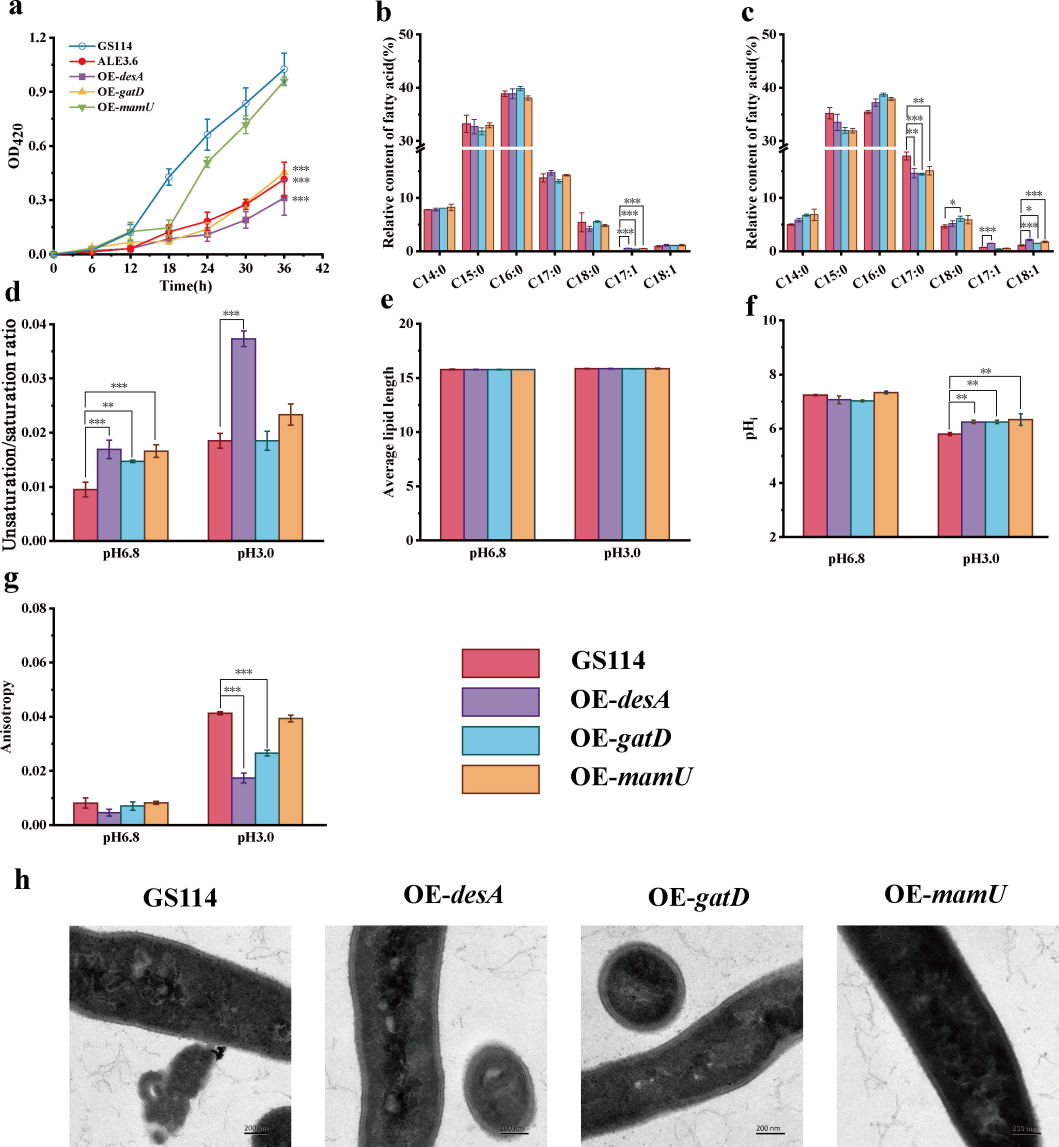
Increased expression of key genes enhanced cell membrane integrity and fluidity of *S. albulus* strains under low pH conditions. (**a**) The effect of low pH environment on the integrity of the cell membrane of each strain of *S. albulus* was measured by the absorbance of the reaction mixture of o-nitrophenyl-β-D-galactopyranoside at 420 nm after low pH treatment. (**b**) Fatty acid contents in the cell membranes of strains GS114, OE-*desA*, OE-*gatD*, and OE-*mamU* after treatment at pH 3.0. (**c**) The fatty acid content in the cell membrane of strains GS114, OE-*desA*, OE-*gatD*, and OE-*mamU* after treatment at pH 3.0. (**d**) Unsaturated:saturated ratio of cell membrane fatty acids of strains GS114, OE-*desA*, OE-*gatD*, and OE-*mamU* after treatment at pH 6.8 and 3.0. (**e**) Average chain length of cell membrane fatty acids of strains GS114, OE-*desA*, OE-*gatD*, and OE-*mamU* after treatment at pH 6.8 and 3.0. (**f**) Intracellular pH of strains GS114, OE-*desA*, OE-*gatD*, and OE-*mamU* after treatment under pH 6.8 and 3.0. (**g**) Membrane fluidity of strains GS114, OE-*desA*, OE-*gatD*, and OE-*mamU* after treatment under pH 6.8 and 3.0. (**h**) TEM images of strains GS114, OE-*desA*, OE-*gatD*, and OE-*mamU* after treatment under pH 3.0. Error bars indicate the standard deviations. *, *P* ≤ 0.05; **, *P* ≤ 0.01; ***, *P* ≤ 0.001.

To further investigate the roles of the key genes *desA*, *gatD*, and *mamU* in enhancing low pH tolerance, we performed an o-nitrophenyl-β-D-galactopyranoside assay to assess the membrane integrity of the overexpressing mutant strains following acid treatment. The final absorbance at 420 nm of strains ALE3.6, OE-*desA*, OE-*gatD*, and OE-*mamU* decreased by 59.5%, 69.5%, 55.9%, and 6.3%, respectively, compared to GS114 (Fig. 5a). These results indicate that the overexpressed strains maintained membrane integrity for a longer time under low pH conditions. We then measured the cell membrane fatty acid content to determine whether these overexpression strains could exhibit similar physiological changes as the mutant strain ALE3.6. After treatment at pH 6.8, the saturated fatty acid content of OE-*desA*, OE-*gatD*, and OE-*mamU* strains did not change significantly compared with GS114, but trace amounts of C_17:1_ that were not found in GS114 appeared in the membranes of these overexpression strains (Fig. 5b). At pH 3.0, the OE-*desA* strain exhibited an 18.0% reduction in C_17:0_ fatty acids, no significant change in C_18:0_, and substantial increases of 161.9% and 96.1% in C_17:1_ and C_18:1_, respectively, compared to GS114. In the OE-*gatD* strain, C_17:0_ showed a 19.0% decrease, no significant alteration in C_17:1_, and increases of 31.1% and 35.0% in C_18:0_ and C_18:1_, respectively. Additionally, the OE-*mamU* strain demonstrated a 15.4% decline in C_17:0_ and a 61.1% rise in C_18:1_, while C_18:0_ and C_17:1_ levels remained unaffected (Fig. 5c). Under pH 6.8 condition, the OE-*desA*, OE-*gatD,* and OE-*mamU* each exhibited significant increases in the fatty acid unsaturation/saturation ratio by 77.9%, 57.9%, and 78.9%, respectively, compared to the strain GS114. At pH 3.0, the fatty acid unsaturation:saturation ratio of the OE-*desA* surged by 101.6% relative to GS114. In contrast, the ratio of OE-*gatD* and OE-*mamU* strains did not show notable alterations under pH 3.0 condition (Fig. 5d). No significant differences in average fatty acid chain length were detected among the strains (Fig. 5e). We also measured pHi using a fluorescence probe. Under pH 6.8, the pHi of all strains remained stable at approximately 7.3. At pH 3.0, the pHi of GS114, measured using a fluorescent probe, was 5.81 ± 0.06, while the pHi of the strains OE-*desA*, OE-*gatD*, and OE-*mamU* were 6.25 ± 0.06, 6.25 ± 0.06, and 6.34 ± 0.2, respectively. Statistical analysis showed that the differences in pHi between the overexpression strains and GS114 were all significant, with *P*-values less than 0.01 (Fig. 5f). Similarly, we employed the DPH fluorescence probe to evaluate changes in membrane fluidity. At pH 6.8, the OE-*desA*, OE-*gatD*, and OE-*mamU* strains did not exhibit significant differences in membrane fluidity compared to the GS114 strain. However, after exposure to pH 3.0, the membrane fluidity of the OE-*desA* and OE-*gatD* strains surged by 141.2% and 54.1%, respectively, compared to the GS114, while the OE-*mamU* strain showed no notable alteration (Fig. 5g). Finally, TEM analysis revealed that the OE-*desA*, OE-*gatD*, and OE-*mamU* strains maintained intact cell morphology under low pH conditions, with no evidence of cell wall damage or cytoplasmic leakage (Fig. 5h). These findings highlight that the upregulation of *desA*, *gatD*, and *mamU* genes is vital for enhancing the low pH tolerance of *S. albulus*, with *desA* exhibiting the most significant impact.

## DISCUSSION

In this study, we obtained a mutant strain, ALE3.6, through adaptive laboratory evolution via continuous passaging. Fermentation and physiological assays confirmed that this strain exhibited significantly enhanced low pH tolerance compared to the parental strain GS114. To identify the key genes involved in the increased low pH tolerance of the mutant, we performed resequencing and qRT-PCR analysis, which identified the genes *desA*, *gatD*, and *mamU*, all of which are involved in the synthesis of the cell wall and cell membrane in gram-positive bacteria. Based on these analyses, we employed reverse engineering to investigate these genes further, confirming that they are critical factors contributing to the enhanced low pH tolerance of the parental strain GS114.

*Streptomyces albulus* underwent adaptive laboratory evolution, successfully isolating a mutant strain, ALE3.6, which can tolerate a pH of 3.6. Traditional strategies such as adaptive evolution, mutagenesis, and co-culture (21–23) have been widely used to improve bacterial resistance to challenging environments. These strategies have successfully produced microbial strains that are resistant to high salinity (24), extreme pH (25), and other chemical stresses (22). Previously, a study reported that the adaptive evolution of *Streptomyces diastatochromogene*s T17 increased ε-PL production by 12.5%, with a final production of 1.1 g/L, and significantly improved cell dry weight compared to the original strain (26). Additionally, adaptive evolution through the environmental autoacidification of *S. albulus* QLU58 has been studied (14). However, these studies did not achieve the best results. In our study, we gradually lowered the pH during ALE fermentation and eventually screened out the mutant strain ALE3.6, which can grow rapidly at pH 3.6 and efficiently produce ε-PL at pH 3.7. While optimizing the fed-batch fermentation process, we observed that strain ALE3.6 accumulated excessive biomass when the fermentation pH exceeded 3.9. A significant increase in sugar consumption rate and oxygen limitation accompanied this biomass accumulation. These findings provide a basis for further investigation of the low pH tolerance of *S. albulus* in future research.

Through whole-genome resequencing and qRT-PCR analysis, key genes associated with enhanced low pH tolerance in mutant ALE3.6 were identified, including *desA*, *gatD*, and *mamU*. Similar studies have recently utilized adaptive evolution and whole-genome resequencing to pinpoint key genes (27–29). However, unlike those studies, we focused our gene sequencing efforts on a single significantly mutated strain, which presented challenges in analyzing the SNP mutation sites. Although we did not directly identify genes associated with low pH tolerance among these mutations, we cannot rule out their indirect impact on the bacterial low pH tolerance mechanism. We plan to explore the SNPs in the mutant strain ALE3.6 in future studies. We identified 10 genes with potential relevance to low pH tolerance from the copy number variation analysis. Further qRT-PCR analysis helped identify three key mutated genes. Interestingly, *desA* is an orthologous gene to one identified in our study of *S. albulus* M-Z18. When the *desA* gene was overexpressed in M-Z18, batch fermentation results showed a 21.1% increase in DCW, but no significant improvement in ε-PL production compared to M-Z18 (13). In contrast, the overexpression of *desA* in GS114 resulted in a significant increase in both final DCW and ε-PL production, which were 104.0% and 169.0% higher, compared to GS114 in this study. We speculate that the difference in the experiments is because strain M-Z18 has a higher low pH tolerance than GS114 (17). Therefore, we believe that *desA* plays a more important role in the low pH tolerance of strain GS114 compared to strain M-Z18.

**Fig 6:**
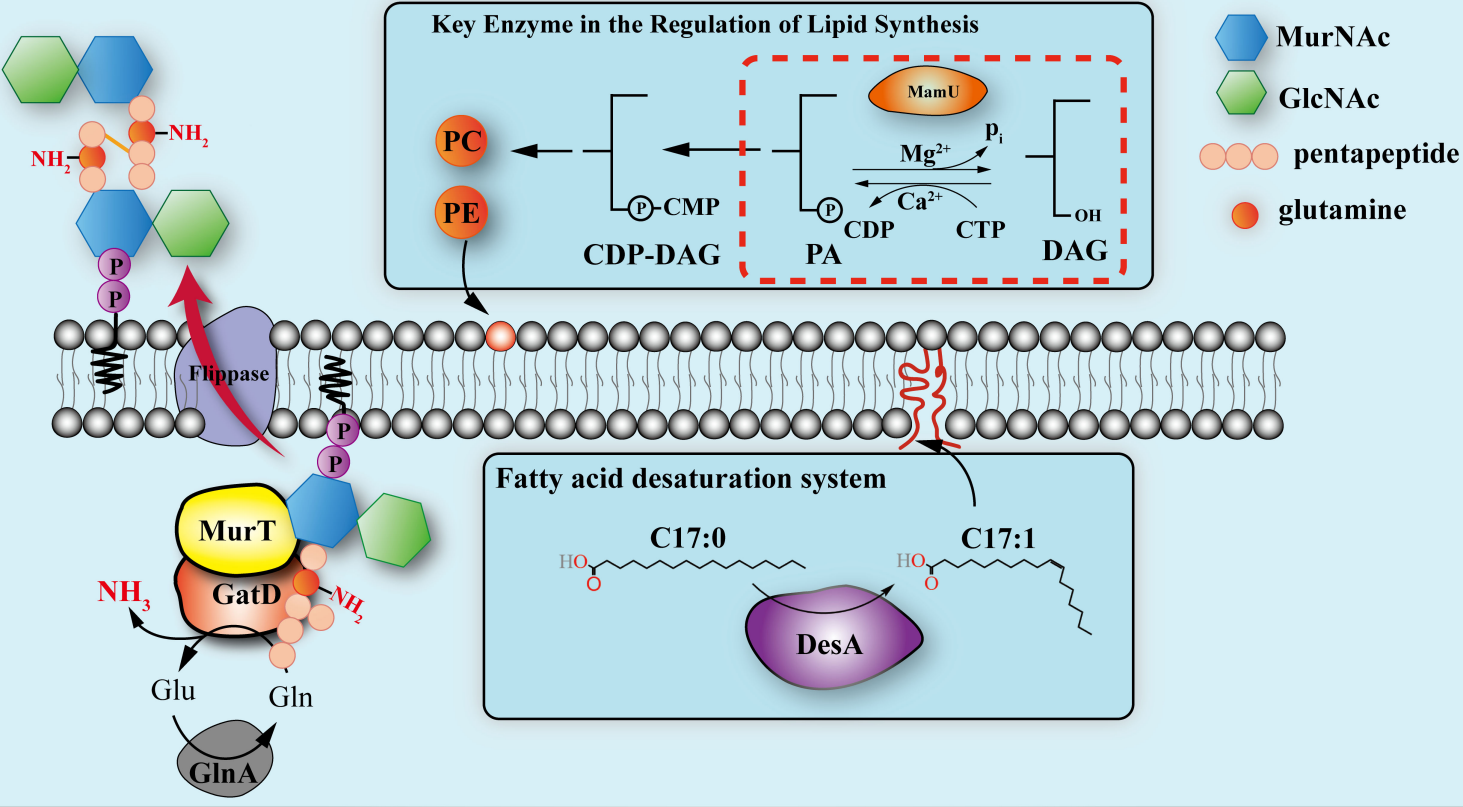
Partial mechanisms of the cell membrane and wall contributing to low pH tolerance in *S. albulus*.

*S. albulus* maintains its survival in a low pH environment by changing the composition of cell membrane fatty acids and enhancing the synthesis of cell walls and cell membrane precursors. This is consistent with recent studies, which suggest that microbial resistance to extreme environments requires a range of genetic and physiological changes (30, 31). Our study indicates that bacteria may adopt multifaceted strategies in their cell membrane and wall composition to cope with low pH conditions. Several factors, including fatty acid chain length (32), unsaturation (33), and phospholipid composition (34), influence membrane fluidity. For example, Δ10(E)-SD can enhance the survival rate and growth speed of fungal strains under low temperature and high salinity (35), while in cyanobacteria, the cold-induced expression of fatty acid desaturase (gene *des*) increases fatty acid unsaturation at low temperatures, thereby enhancing cold and salt tolerance (36). In *Saccharomyces cerevisiae*, the overexpression of desaturase genes OLE1 and FAD2 from *Rhodotorula* improves stress resistance by enhancing membrane fluidity and integrity (37). In our study, overexpression of the *desA* gene led to an increased content of unsaturated fatty acids in the cell membrane. Surprisingly, the increase in membrane fluidity did not impair low pH tolerance (38), suggesting that conversion of saturated fatty acids C_17:0_ to C_17:1_ and increased membrane fluidity help *S. albulus* survive in low pH environments. Overexpression of the genes *yvhB* and *xynD*, which have the same function as the MurT/GatD complex in catalyzing MurNAc O-acetylation and GlcNAc N-deacetylation, improved the cell wall integrity and acid resistance of *Lactococcus lactis* (39). GatD is a glutaminase, which provides ammonia for the amination reaction of MurT, generates ammonia by hydrolyzing L-glutamate, and then transfers ammonia to MurT through a channel (40). Physiological analysis experiments can prove that increased *gatD* expression can help bacteria provide more ammonia for cell wall repair and maintain intracellular pH. Finally, MamU is a bifunctional phosphatase PAP2/diacylglycerol kinase family protein that plays a key role in maintaining the balance of cell membrane phospholipids, affecting key steps in triglyceride synthesis and ultimately enhancing tolerance to abiotic stresses (41). MamU also functions as a pH biosensor, directly influencing gene expression and regulating phospholipid metabolism based on nutrient availability, thereby controlling membrane biosynthesis pathways (42). We found that increased metabolism of phosphatidic acid and expression of bifunctional enzymes involved in its synthesis in *S. albulus* contribute to survival in low pH environments and stabilization of pHi. Based on these results, we summarized the partial response mechanisms of the *S. albulus* cell membrane and cell wall under low pH conditions (Fig. 6). Through annotating and Gene Ontology (GO) enrichment of resequencing data (Fig. S1e), we found that the copy number of multiple transcriptional regulator genes was increased (e.g., *cutR*, *cutS*, *afsQ_1_/afsQ_2_*, *cseC*). A comprehensive exploration of the global regulatory factors involved in low pH tolerance and a detailed analysis of the dynamic regulatory mechanisms of *S. albulus* under low pH stress combined with transcriptomics will be the focus of our future research.

In conclusion, the reverse engineering of the ALE mutant establishes a foundation for investigating the mechanisms of low pH tolerance in *S. albulus*. It highlights the regulatory changes in genes associated with the cell membrane and wall in response to acidic environments. The findings indicate that the upregulation of genes such as *desA*, *gatD*, and *mamU* contributes to enhanced survival and increased ε-PL synthesis rate in low pH conditions. This research significantly advances our understanding of low pH tolerance in *Streptomyces*.

## MATERIALS AND METHODS

### Strains and culture conditions

*Streptomyces albulus* GS114 served as the initial strain for ALE. Table 1 details the strains and plasmids employed in this study. *Escherichia coli* was cultured in Luria-Bertani medium. For spore cultivation, *S. albulus* was grown in BTN medium (10 g/L glucose, 1 g/L yeast extract, 2 g/L fish meal peptone, pH 7.5). The spores were then inoculated into M3G medium (50 g/L glucose, 5 g/L yeast extract, 10 g/L (NH_4_)_2_SO_4_, 1.36 g/L KH_2_PO_4_, 0.8 g/L K_2_HPO_4_·3H_2_O, 0.5 g/L MgSO_4_·7H_2_O, 0.04 g/L ZnSO_4_·7H_2_O, 0.03 g/L FeSO_4_·7H_2_O, pH 6.8) and fermented at 30°C and 200 rpm for 24 h to generate the seed culture. This seed culture was subsequently transferred at an 8% inoculation ratio into YP medium (60 g/L glucose, 10 g/L yeast extract, 10 g/L (NH_4_)_2_SO_4_, 4 g/L KH_2_PO_4_, 0.5 g/L MgSO_4_·7H_2_O, 0.04 g/L ZnSO_4_·7H_2_O, 0.03 g/L FeSO_4_·7H_2_O, pH 6.8). YP medium was utilized as the fermentation medium for cultivation in shake flasks and 1 L and 5 L bioreactors. For intergeneric conjugation between *E. coli* ET12567 and *S. albulus* GS114, MS medium supplemented with 10 mM MgCl_2_ and containing soybean powder (20 g/L), D-mannitol (20 g/L), and agar (20 g/L) was employed.

**TABLE 1:** Strains and plasmids used in this study

Strain or plasmid	Relevant genotype or characteristic	Reference
*Streptomyces albulus* strains		
GS114	Mutant of M-Z18 by DES, ARTP, and protoplast fusion	Our laboratory
OE-*desA*	GS114 harboring pIB139-*desA*	This study
OE-*gatD*	GS114 harboring pIB139-*gatD*	This study
OE-*mamU*	GS114 harboring pIB139-*mamU*	This study
Anti-*desa*	GS114 harboring asRNA-*desA*	This study
Anti-*gatd*	GS114 harboring asRNA-*gatD*	This study
Anti-*mamu*	GS114 harboring asRNA-*mamU*	This study
*E. coli* strains		
DH5α	General cloning host	Takara
ET12567/pUZ8002	Donor strain for conjugation between *E. coli* and *Streptomyces*	(43)
Plasmids		
pIB139	Integrative overexpression plasmid, Apr, p_ermE_	(44)
pIB139-*hfq-micc-to*	Antisense RNA plasmid	Our laboratory
pIB139-*desA*	p_ermE_-*desA*	This study
pIB139-*gatD*	p_ermE_-*gatD*	This study
pIB139-*mamU*	p_ermE_-*mamU*	This study
asRNA-*desA*	P_micc_+sRNA-*desA*	This study
asRNA-*gatD*	P_micc_+sRNA-*gatD*	This study
asRNA-*mamU*	P_micc_+sRNA-*mamU*	This study

### Adaptive laboratory evolution

We cultured *S. albulus* GS114 spores for 24 h in seed culture. The seed culture was inoculated into M3G medium at an 8% ratio with an initial pH of 4.0. The inoculated medium was incubated at 30°C and 200 rpm for 36 h. Subsequently, the culture was transferred to a fresh medium with the same inoculation ratio. To apply selective pressure for adaptive evolution, we gradually decreased the pH by 0.2 once cell growth stabilized. At each pH level, colonies that demonstrated rapid growth and high sporulation on M3G solid plates at pH 4.0 were selected as evolved strains with enhanced low pH tolerance.

### Cultivation and fermentation

In this study, *S. albulus* spores were cultured on BTN medium at 30°C for 5–6 days. After incubation, the spores were inoculated into an M3G medium and cultured at 30°C, with shaking at 200 rpm for 24 h to obtain the necessary seed culture. Fermentation experiments were then conducted in 1 L and 5 L bioreactors following the methodology described in reference 45, with minor modifications.

To treat the bacteria with low pH, 10 mL of the seed solution was added to the fermentation medium adjusted to pH 2.5, so that the final pH was about 3.0. The bacteria were incubated at 30°C and 200 rpm for 1.5 h. Subsequently, the fermentation broth was centrifuged at 12,000 rpm and 4°C for 1 minute to harvest the low pH-treated bacterial cells for further experiments.

Fed-batch fermentation was performed in a 1 L bioreactor (Fig. S2). Ammonia (18%, wt/vol) maintained the pH at either 4.0 or 3.7 based on experimental requirements. Dissolved oxygen levels were kept between 25 and 30% through rotational speed and aeration. Residual glucose concentration was monitored, and when it reached 10 g/L, additional glucose was supplemented to maintain levels between 10 and 15 g/L. The fermentation was concluded after 60 h.

Fed-batch fermentation was performed in a 5 L bioreactor. For the GS114 strain, ammonia (18%, wt/vol) maintained the pH at 6.0 ± 0.1 for 7.5 h during pre-culturing. Following the pH shock, ammonia was used to maintain the pH at 4.0 ± 0.25. Dissolved oxygen levels were controlled between 25 and 30% through rotational speed and aeration (Fig. S3a). When the glucose concentration decreased to 10 g/L, additional glucose was added to maintain concentrations between 10 and 15 g/L. For the mutant strain ALE3.6, the pre-culture phase was omitted. Following the pH shock, ammonia maintained the pH at 3.7 ± 0.1, while all other control conditions remained identical (Fig. S3b). The fermentation was concluded after 96 h.

### Whole-genome resequencing

The ALE3.6 strain was cultured at 30°C with shaking at 200 rpm for 24 h. The culture was then centrifuged at 4°C, and the supernatant was discarded. The pellet was washed twice with physiological saline. The final pellet was flash-frozen in liquid nitrogen and sent to Genedenovo Biotechnology for whole-genome resequencing analysis. DNA libraries were sequenced using the Illumina sequencing platform by Genedenovo Biotechnology (Guangzhou, China). Genomic DNA was extracted from the strain and randomly fragmented into short segments using enzymes, followed by end repair. dA tails were added to the DNA fragments, and sequencing adapters were ligated. The adapter-ligated DNA fragments were purified with AMPure XP magnetic beads. Fragments ranging from 300 to 400 bp were selected for PCR amplification. The constructed library was purified, quality-checked, and sequenced on the HiSeq X10 PE150 platform. After data filtering, genome alignment was performed to analyze SNPs, InDels, and CNVs in the ALE3.6 strain. Copy number variation analysis was conducted using Cnvpytor, which normalized variant copy numbers based on established formulas and eliminated measurement errors (46).

### Recombinant strain construction

Primers were designed to amplify the target gene segments through PCR, after which the amplified fragments were purified and inserted into plasmid pIB139, resulting in the creation of pIB139-*desA*, pIB139-*gatD*, and pIB139-*mamU*. The primers used are listed in Table 2. For the gene silencing experiments, the PermE, *hfq*, and *micc* regions were PCR-amplified from the genome of *Escherichia coli* E12, while the transcription terminator *to* region was amplified from the pAcas9 plasmid available in our laboratory. The P_ermE_ and *hfq* fragments were ligated into the *EcoR* I-digested, linearized pIB139 vector using a one-step cloning kit, resulting in the recombinant plasmid pIB139-*hfq*. Subsequently, the *micc* and *to* fragments were inserted into the pIB139-*hfq* vector treated with *Nde* I and *EcoR* V, yielding the recombinant plasmid pIB139-*hfq-micc-to*. Then, 24 bp antisense fragments of *desA*, *gatD*, and *mamU* were amplified from *S. albulus* GS114. Finally, these 24 bp antisense fragments were ligated into the *Nde* I-treated pIB139-*hfq-micc-to* vector to construct the final recombinant plasmid. Following the protocol detailed in a prior study (47), these plasmids were transferred into *S. albulus* GS114 via intergeneric conjugation facilitated by *Escherichia coli* ET12567/pUZ8002.

**TABLE 2:** Primers used in this study

Function and primer	Sequence (5′–3′)
Point mutation	
*desA*-F1	GGTTGGTAGGATCCACATATGTTCCCTCGTGACCATCGCC
*desA*-F2	CATGATTACGAATTCGATATCTCGAAGCGGCACTCAGCC
*gatD*-F1	GGTTGGTAGGATCCACATATGTCAGACGTTCTGCGTCGCC
*gatD*-F2	CATGATTACGAATTCGATATCATGAGTGACAACAGCCTGCGC'
*mamU*- F1	CATGATTACGAATTCGATATCTCAGGCGGGAAGGAGGCG
*mamU* - F2	GGTTGGTAGGATCCACATATGCCCATGCGAACGCCAACG
Antisense RNA inhibition	
anti-*desA*-F1	CTAGAGGATCCCCAACATATGAAAAAAAAAGCCCGGACGA
anti-*desA*-F2	GGTTGGTAGGATCCACATATGAGGCGGTCGGGGGCGATGGTCACGTTATATGCCTTTATTGTCACAGATTTTA
anti-*gatD*-F1	CTAGAGGATCCCCAACATATGAAAAAAAAAGCCCGGACGA
anti-*gatD*-F2	GGTTGGTAGGATCCACATATGCACGCGCAGGCTGTTGTCACTCATGTTATATGCCTTTATTGTCACAGATTTTA
anti-*mamU*-F1	CTAGAGGATCCCCAACATATGAAAAAAAAAGCCCGGACGA
anti-*mamU*-F2	GGTTGGTAGGATCCACATATGGGTGAGGAGCGTTGGCGTTCGCATGTTATATGCCTTTATTGTCACAGATTTTA
qRT-PCR	
*desA*-F1	ATCGCCAACGACGAGAAC
*desA*-F2	ATCCGCAGGTTGTAGATCC
*gatD*-F1	CGCCGATCAGGTAGATGTC
*gatD*-F2	GCGTGGTGTGGATCTATCC
*adiC*-F1	CGTACTGGACCATGACCT
*adiC*-F2	AGCGGCACGAACTTCA
*tesB*-F1	AGGAGGCGGTCAGATTG
*tesB*-F2	CTGCTGGACCTGGAACA
*oxyS*-F1	GACCGTGCTGGAACAAC
*oxyS*-F2	TCCTCCAACTCCGTTATCA
*oxyE*-F1	CTCTGCTGCGACTTCTG
*oxyE*-F2	CTGCTGGAGGTCACCTT
*otcC*-F1	TCAACCTCGGCTGGAAG
*otcC*-F2	GTCGTAGCGGATGAGTTC
*fabF*-F1	GGAGACCGCCTTCATCT
*fabF*-F2	GTTCTACGACCAGCAGAG
*uvrA*-F1	TGGCATCGACGTGGACATC
*uvrA*-F2	ATCCGCAGGGTGTTGGAGA
*mamU*-F1	GACGAACAGCAGCCAGAT
*mamU*-F2	GCCAACACTTCCTCAACAC

### Measurement of ε-PL production and DCW

The fermentation broth was centrifuged at 12,000 × g for 10 minutes. ε-PL production in the supernatant was measured using the methyl orange method (48). The pellet was washed twice with deionized water and dried at 105°C until a constant weight was achieved to determine dry cell weight (44). All measurements were performed in triplicate.

### Spot assay

Spores from different strains were scraped (four loops) into shake flasks containing glass beads and shaken to obtain a spore suspension. The spore suspension of each strain was measured at 600 nm using a UV spectrophotometer, and the OD_600_ was uniformly adjusted to 0.4 as the original solution for serial dilution (10^0^). The spore suspensions of each strain were serially diluted (10^−1^, 10^−2^, 10^−3^, 10^−4^, 10^−5^, 10^−6^) and spot-plated onto M3G plates at pH 6.0 and pH 3.7. The plates were incubated statically at 30°C for 7 days, and colony growth was observed.

### qRT-PCR analysis

The *S. albulus* fermentation broth, following acid treatment, was immediately frozen in 99.99% liquid nitrogen. Total RNA was subsequently extracted using a microbial RNA extraction kit (Medtech). First-strand cDNA was synthesized from 1 µg of total RNA using the PrimeScript II 1st Strand cDNA Synthesis Kit (TaKaRa). The resulting cDNA mixture was diluted to approximately 100 ng/μL and used as a template for gene expression analysis. qRT-PCR was performed using the iQ5 real-time detection system (Bio-Rad, Hercules, CA) and SYBR Premix Ex Taq (TaKaRa, Japan). All reactions were conducted in triplicate, and the relative gene expression was analyzed using the 2^−△△Ct^ method, with the housekeeping gene *hrdB* as the endogenous control (49).

### Analysis of cell membrane integrity and fluidity

Take 5 mL of the acid-treated fermentation broth and wash it twice with pre-cooled 10 mM phosphate-buffered saline (PBS) buffer. Resuspend the cells in PBS buffer and measure and dilute the cell suspension to achieve a uniform OD_600_ of 1.0 for each strain. Finally, 5 mL of the resuspended cells was transferred into 10 mM PBS buffer containing a final concentration of 3 mg/mL o-nitrophenyl-β-D-galactopyranoside (ONPG) and incubated at 30°C, with shaking at 200 rpm for 36 h. Samples of the reaction mixture are taken every 6 h to measure the absorbance at 420 nm (50–52).

Five milliliters of fermentation broth was centrifuged at 10,000 rpm for 8 minutes, and the supernatant was discarded. The resulting cell pellet was washed twice with 10 mM PBS buffer containing 0.25% (vol/vol) glutaraldehyde. The cells were then resuspended in a 10 mM PBS solution containing 0.1% (vol/vol) tetrahydrofuran, 0.25% (vol/vol) glutaraldehyde, and a final concentration of 3 × 10^−5^ M DPH. Unlabeled samples were dissolved without affecting light intensity. Fluorescence measurements were taken at an excitation wavelength of 360 nm, emission wavelength of 430 nm, and a slit width of 5 nm. The fluorescence anisotropy value was calculated using the method described. Finally, cell membrane fluidity was analyzed based on the negative correlation with fluorescence anisotropy (53).

### Fatty acid extraction and analysis

The mycelium was harvested by centrifugation at 3,000 × *g* for 15 minutes and washed three times with distilled water (54). A total of 40–50 mg of mycelium (wet weight) was subjected to saponification and methylation. The resulting methyl ester mixture was analyzed using GC-MS (TSQ 8000, triple quadrupole, Thermo Fisher Scientific, USA). The specific analytical procedures were performed as described in the referenced method. The cell membrane fatty acids finally determined were myristic acid (C_14:0_), pentadecanoic acid (C_15:0_), hexadecanoic acid (C_16:0_), dodecanoic acid (C_17:0_), stearic acid (C_18:0_), oleic acid (C_18:1_), and cis-10-dodecenoic acid (C_17:1_).

### Intracellular pH measurement

The intracellular pH was measured according to the method reported by Breeuwer (55), with slight modifications. The specific process is as follows: the fermentation broth was centrifuged and washed three times with PBS buffer (4,500 × *g*, 10 minutes), and the supernatant was discarded. A total of 0.5 g of wet mycelium was resuspended in HEPES-K (pH 8.0) solution and sonicated in an ice-water bath for 5 minutes (power 60%, 2 s on/2 s off). The suspension was centrifuged at 600 × *g* for 4 minutes to remove unbroken mycelium, and the supernatant was discarded. The pellet was resuspended in HEPES-K (pH 8.0) solution, thoroughly mixed, and 1 µL of BCECF-AM was added. The mixture was incubated at 30°C in a water bath for 20 minutes, then centrifuged and washed three times with PBS buffer (7,000 × *g*, 30 s), and resuspended in PBS buffer. The fluorescence intensity of the cell suspension (Itotal) and the supernatant (Ifiltrate) was measured with excitation wavelengths of 490 nm and 440 nm and an emission wavelength of 525 nm. The measurement volume was 200 µL, and the slit width was set at 9 nm. All PBS buffer solutions used were pre-cooled at 4°C, and centrifugation was also performed at 4°C. Unless otherwise specified, all operations were conducted on ice. Finally, the fluorescence intensity was calculated according to the formula in the reference (48).

### Electron microscopy analysis

The low pH-treated mycelia were washed twice with 10 mM PBS containing 2.5% glutaraldehyde, then resuspended in the same solution and fixed at room temperature for 2 h. The samples were then stained, embedded, and sectioned by Servicebio (China) before observation of the cell cross-sections under a transmission electron microscope (H-7650).

### Statistical analysis

All experiments were performed in triplicate, and the data are expressed as mean ± standard deviation. Statistical analysis was performed using SPSS (version 22.0, SPSS Inc., Chicago, IL, USA). One-way analysis of variance was used, followed by the Tukey’s *post hoc* test for pairwise comparisons. *P* values were calculated, and the significance levels are indicated in the figures as follows: * for *P* ≤ 0.05, ** for *P* ≤ 0.01, and *** for *P* ≤ 0.001.

## Data Availability

Whole-genome sequencing data for *S. albulus* ALE3.6 have been submitted to the SRA database under accession number SRR31845403.

## SUPPLEMENTAL MATERIAL

Supplemental figures aem.00036-25-s0001.docx Figures S1 to S3.

ASM does not own the copyrights to Supplemental Material that may be linked to, or accessed through, an article. The authors have granted ASM a non-exclusive, world-wide license to publish the Supplemental Material files. Please contact the corresponding author directly for reuse.
