# Supplementary material for: Adaptive evolution and reverse engineering to explore the low pH tolerance mechanisms of Streptomyces albulus
Source: Appl Environ Microbiol. 2025 Mar 31;91(4):e00036-25. doi: 10.1128/aem.00036-25 (PMC12016549; doi:10.1128/aem.00036-25)
Supplement: Supplemental figures — Figures S1 to S3. [file aem.00036-25-s0001.docx]

**Adaptive evolution and reverse engineering to explore the low pH tolerance mechanisms of *Streptomyces albulus***

Yuxi Liu, Tianyi Liu, Yulin Zhang, Liang Wang, Hongjian Zhang, Jianhua Zhang, Xusheng Chen*

Key Laboratory of Industrial Biotechnology, Ministry of Education; School of Biotechnology, Jiangnan University, Wuxi 214122, China

^*^Correspondence to: Prof. Chen, School of Biotechnology, Jiangnan University, 1800 Lihu Road, Wuxi 214122, Jiangsu, China

Tel & Fax: 0086-510-85918296

E-mail: chenxs@jiangnan.edu.cn


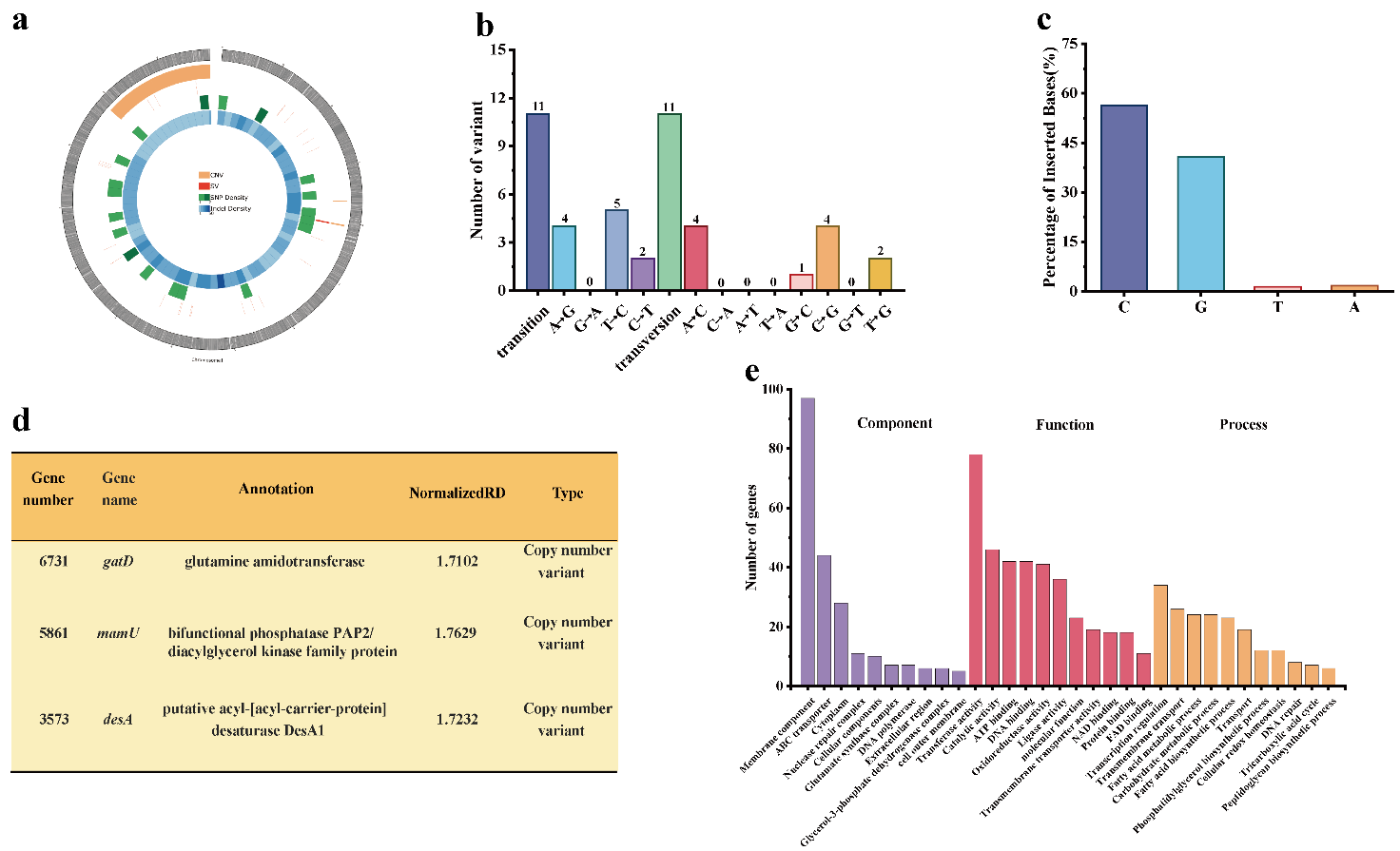
Fig. S1: (a) A circos plot uses multiple concentric rings to display various layers of genomic information, including CNV, SV, SNP density, and Indel density. (b) Statistical analysis of the number of SNP transition and transversion mutations in the mutant strain ALE3.6. (c) Proportion of each nucleotide base in insertion mutations from the InDel analysis of the mutant strain ALE3.6. (d) Key genes with copy number variations are identified in whole-genome resequencing, where normalizedRD represents system-normalized data. The calculation of normalizedRD was performed as described in Materials and Methods (46) (e) GO functional enrichment analysis of differentially expressed genes in strain ALE3.6.


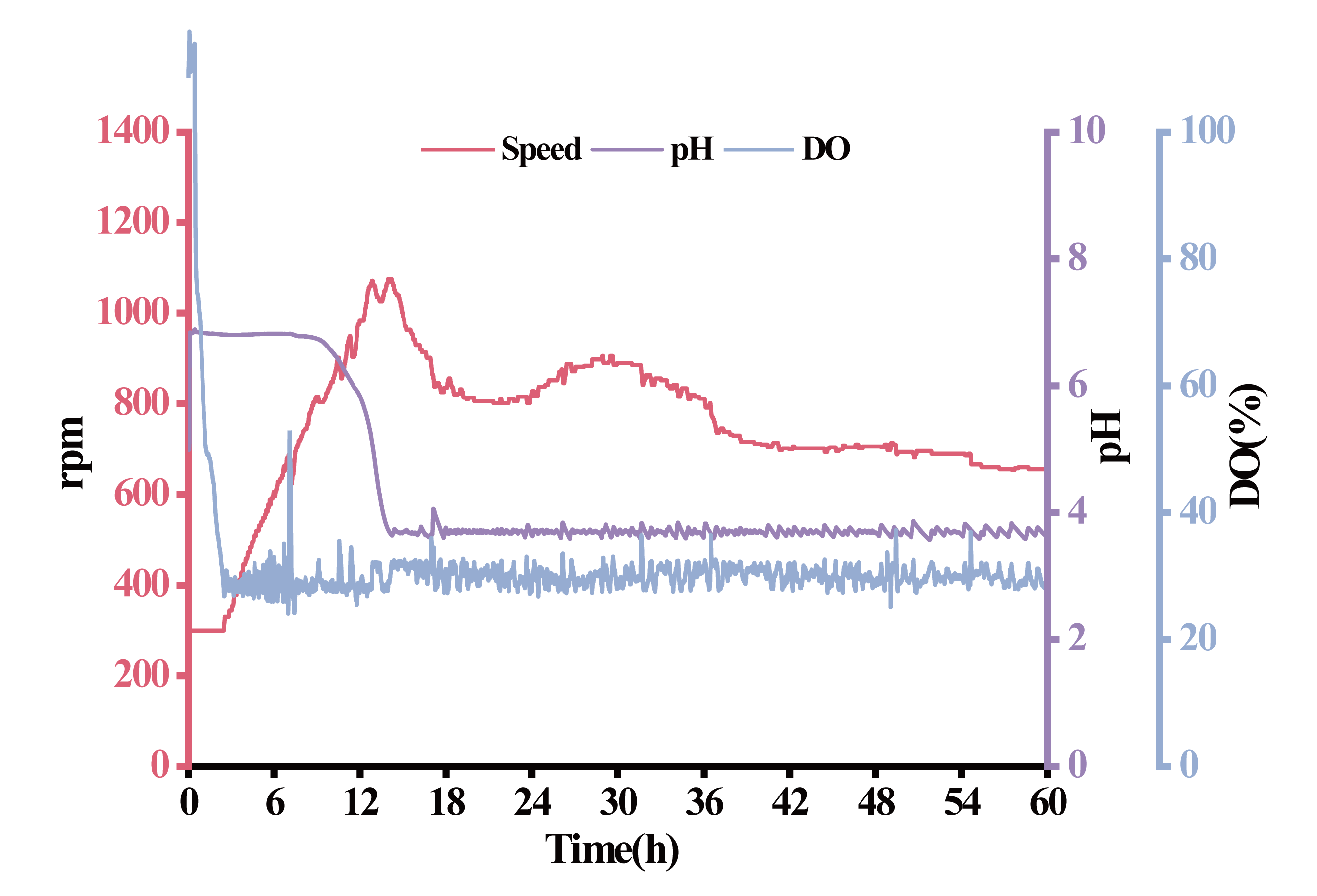
Fig. S2: Process parameters for 60-hour fed-batch fermentation using a constant pH strategy in a 1 L parallel bioreactor. (The figure illustrates the fermentation process of GS114 as an example; other fermentation processes were similar and are not shown.)


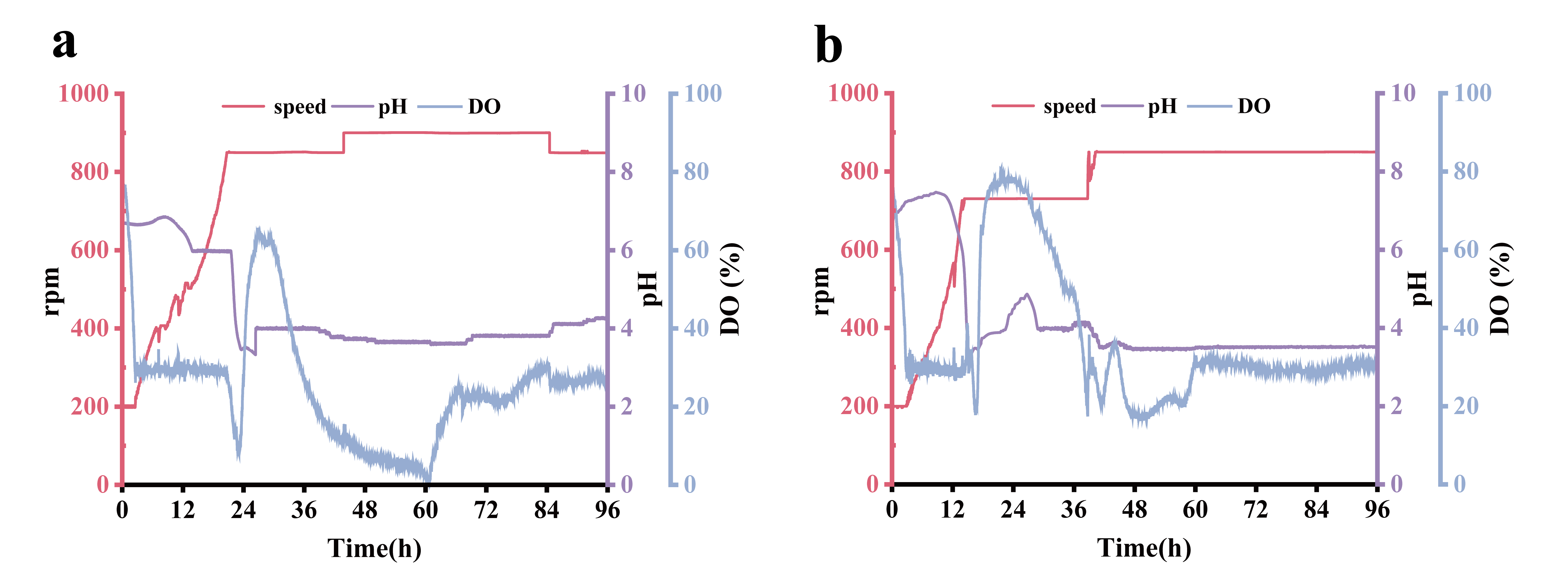
Fig. S3: Process parameters during 96-hour fed-batch fermentation based on a pH shock strategy in a 5 L bioreactor. (a) GS114 strain with pH regulated at 4.0 ± 0.25, including pH, agitation speed, and dissolved oxygen. (b) Mutant strain ALE3.6 with pH regulated at 3.7 ± 0.1, including pH, agitation speed, and dissolved oxygen.
